# The Coronary Arteries in Adults after the Arterial Switch Operation: A Systematic Review

**DOI:** 10.3390/jcdd8090102

**Published:** 2021-08-26

**Authors:** Leo J. Engele, Barbara J. M. Mulder, Jan W. Schoones, Philippine Kiès, Anastasia D. Egorova, Hubert W. Vliegen, Mark G. Hazekamp, Berto J. Bouma, Monique R. M. Jongbloed

**Affiliations:** 1 Center for Congenital Heart Disease Amsterdam-Leiden (CAHAL), Department of Clinical and Experimental Cardiology, Amsterdam Cardiovascular Sciences, Heart Centre, Amsterdam UMC, University of Amsterdam, 1105 AZ Amsterdam, The Netherlands; l.j.engele@amsterdamumc.nl (L.J.E.); b.j.mulder@amsterdamumc.nl (B.J.M.M.); b.j.bouma@amsterdamumc.nl (B.J.B.); 2 Netherlands Heart Institute, 3511 EP Utrecht, The Netherlands; 3 Directorate of Research Policy, Leiden University Medical Center, 2333 ZA Leiden, The Netherlands; j.w.schoones@lumc.nl; 4 Center for Congenital Heart Disease Amsterdam-Leiden (CAHAL), Department of Cardiology, Leiden University Medical Center, 2333 ZA Leiden, The Netherlands; p.kies@lumc.nl (P.K.); a.egorova@lumc.nl (A.D.E.); h.w.vliegen@lumc.nl (H.W.V.); 5 Center for Congenital Heart Disease Amsterdam-Leiden (CAHAL), Department of Cardiothoracic Surgery, Leiden University Medical Center, 2333 ZA Leiden, The Netherlands; m.g.hazekamp@lumc.nl; 6 Center for Congenital Heart Disease Amsterdam-Leiden (CAHAL), Department of Anatomy and Embryology, Leiden University Medical Center, 2333 ZA Leiden, The Netherlands

**Keywords:** transposition of the great arteries, arterial switch operation, coronary artery, coronary complications, imaging

## Abstract

Coronary artery status in adults long after the arterial switch operation (ASO) is unclear. We conducted a systematic review to provide an overview of coronary complications during adulthood and to evaluate the value of routine coronary imaging in adults after ASO, in light of current guidelines. Articles were screened for the inclusion of adult ASO patients and data on coronary complications and findings of coronary imaging were collected. A total of 993 adults were followed with a median available follow-up of only 2.0 years after reaching adulthood. Myocardial ischemia was suspected in 17/192 patients (8.9%). The number of coronary interventions was four (0.4%), and coronary death was reported in four (0.4%) patients. A lack of ischemia-related symptoms cannot be excluded because innervation studies indicated deficient cardiac innervation after ASO, although data is limited. Anatomical high-risk features found by routine coronary computed tomography (cCT) included stenosis (4%), acute angle (40%), kinking (24%) and inter-arterial course (11%). No coronary complications were reported during pregnancy (n = 45), although, remarkably, four (9%) patients developed heart failure. The 2020 European Society of Cardiology (ESC) guidelines state that routine screening for coronary pathologies is questionable. Based on current findings and in line with the 2018 American ACC/AHA guidelines a baseline assessment of the coronary arteries in all ASO adults seems justifiable. Thereafter, an individualized coronary follow-up strategy is advisable at least until significant duration of follow-up is available.

## 1. Introduction

**Figure 1:**
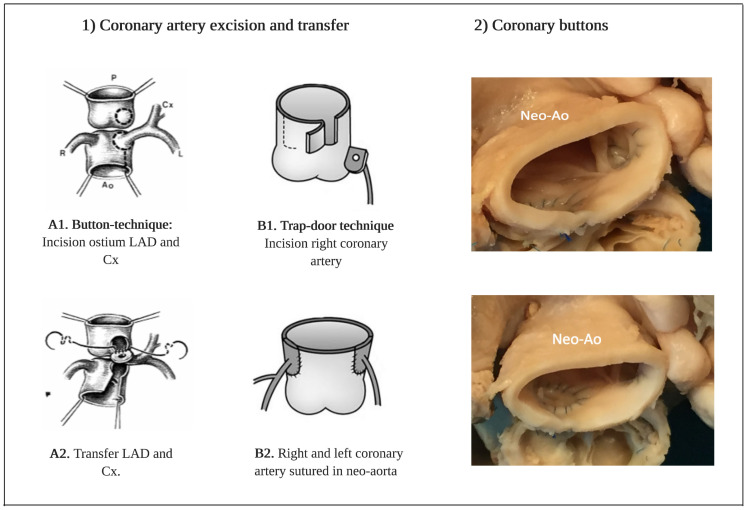
(**1**) Operation techniques for translocation of the coronary arteries during the arterial switch operation. (**A1**,**A2**) Button-technique of coronary artery excision and transfer to the neo-aorta in most common type (1R-2LCx). (**B1**,**B2**) Trap-door technique of coronary artery excision and transfer to the neo-aorta. (**2**) Coronary buttons in the neo-aorta in a patient with transposition of the great arteries after arterial switch. Abbreviations: LAD = left anterior descending artery, Cx = circumflex artery, Neo-Ao = Neo-aorta. Note. Images A1 and A2-1D adapted with approval of the author from the thesis: ‘The Arterial Switch Operation–Rationale, Results, Perspectives, by J.M. Quaegebeur, 1986, Chapter IV, p127, ISBN 90-9001327. Images B1 and B2 are adapted from ‘The importance of neo-aortic root geometry in the arterial switch operation with the trap-door technique in the subsequent development of aortic valve regurgitation’, by Jhang et al. European Journal of Cardio-thoracic Surgery, 2012 Nov; 42(5): 794–92013; 96: 1390–1397. Copyright 2021 by Copyright Clearance Center.

Transposition of the great arteries (TGA) is a congenital heart defect with a prevalence of approximately 4.7 per 10,000 newborns and represents 5% to 7% of all congenital heart disease (CHD) [1,2]. Today, the arterial switch operation (ASO) is the operation of choice for anatomical correction in newborns with TGA and selected cases of double outlet right ventricle (DORV). The ASO is also part of the double switch in congenitally corrected TGA (ccTGA) patients. The ASO was performed successfully for the first time by Jatene and colleagues in 1975 [3,4], and after a transition period in the 1980s, it has now replaced the atrial switch procedures in the vast majority of cases. Translocation of the coronary arteries is the most critical step during the arterial switch procedure. The coronary arteries are excised from the aorta (neo-pulmonary trunk) after which the coronary artery is sutured into the pulmonary artery (neo-aorta) (Figure 1). Several factors contribute to a successful coronary transfer, including a sufficient length of the coronary artery and the coronary anatomy. Coronary events are an important cause of death and most often occur early after ASO; previous studies reported a prevalence of coronary obstruction after ASO from 2% to 11% in children [5,6]. 

Less is known about the long-term patency of the translocated coronary arteries and the occurrence of myocardial ischemia during adulthood. Potential mechanisms for myocardial ischemia after translocation of the coronary arteries include coronary kinking, anatomical distortion, stretching, and extrinsic compression. Furthermore, growth of the neo-aorta and proximal coronary arteries during lifetime may result in an increased risk for anatomical high-risk features of the coronary arteries including acute angle, mechanical kinking or stretching [7,8,9]. Patients with an unusual anatomical coronary pattern may be at higher risk for the occurrence of late coronary complications [10]. Due to cardiac denervation after dissecting the cardiac plexus during ASO, patients with a coronary artery stenosis may be asymptomatic. Previous long-term follow-up studies in children reported significant coronary stenosis after ASO in asymptomatic patients [6,7].

Due to the lifelong risk of coronary complications after the ASO, periodic surveillance of the coronary arteries is recommended by current guidelines [11,12]. Non-invasive examinations for detecting myocardial ischemia with ECG, stress echocardiography, exercise testing, and perfusion scintigraphy have shown low sensitivity for detecting significant coronary stenosis [6]. However, Cardiovascular Magnetic Resonance (CMR) and coronary computed tomography (cCT) can be of value in timely detection of coronary complications. There is currently no consensus regarding the optimal follow-up interval and imaging modality for surveillance of the translocated coronary arteries. 

Currently, the group of young adult ASO patients is growing. Although many studies reported coronary complications during childhood, a structured overview of available literature concerning coronary status in adults is lacking. Therefore, we performed a systematic review to provide an overview of currently available literature reporting on the incidence of coronary complications specifically during adulthood and the value of coronary imaging in adults after ASO. Besides, the effects of pregnancy on coronary outcomes and studies on cardiac innervation, given the potential relevance for symptomatology, were analysed. Findings will be discussed in light of current coronary follow-up recommendations after the ASO.

## 2. Materials and Methods

This systematic review was conducted according to the Preferred Reporting Items for Systematic Reviews and Meta Analyses (PRISMA) checklist [11]. Quality assessment of included long-term follow–up cohort studies was performed with the Newcastle Ottawa Scale (NOS) [13]. Studies were scored on the following two domains: (1) selection, including representativeness of the exposed cohort, ascertainment of exposure, and demonstration that the outcome of interest was not present at the start of the study; (2) outcome, including assessment of outcome, follow-up period, and adequacy of follow-up. The maximum number of awarded stars was six. 

### 2.1. Search Strategy

A comprehensive literature search was performed in PubMed, Embase, Web of Science, Cochrane Library and Emcare. Language was restricted to English and German. Because we focused on the outcomes in adults, only articles which were published between 1994 (18 years after introduction of the ASO) and August 2020 were selected. The search strategy was carried out by using key words for arterial switch, transposition of the great arteries, coronary outcome, long-term outcome, and ischemia. (For complete query: see Appendix A). Duplicate articles were removed.

### 2.2. Selection Criteria

Cohort studies with TGA, DORV or ccTGA patients after ASO were included. Long-term outcome on coronary interventions or coronary death in adults (≥18 years) was retrieved. Because the purpose was to evaluate the long-term coronary status after arterial switch, patients with one-stage repair, two-stage repair, and late arterial switch were all included in analysis. Furthermore, cross-sectional coronary imaging studies investigating coronary anatomy or myocardial perfusion with any imaging modality were considered eligible for the review. Finally, studies reporting cardiac outcomes during pregnancy, cardiac innervation, and case reports on coronary complications were included. Studies reporting other than coronary outcome in adults after ASO, description of outcome exclusively in children (<18 year), or patients with a non-identifiable age were excluded. In addition, reviews, editorials and articles in which the full-text could not be retrieved were excluded. Reference lists of reviews were searched for eligible articles which were not identified in the literature search. 

### 2.3. Definitions

The literature search was restricted to adults (18 years or older) because the purpose was to evaluate the long-term patency of the coronary arteries without focusing on the early and midterm outcome. Coronary complications were defined as percutaneous coronary intervention, coronary surgery or coronary death. The following causes of death were interpreted as coronary related: (aborted) sudden cardiac death or death related to proven or possible ischemic heart failure. All imaging techniques performed in adults to assess coronary anatomy or coronary function were considered eligible for systematic review. When duplication of patient data in studies from the same institutions were suspected, the study with the highest number of ASO adults with description of coronary complications was included. 

### 2.4. Data Extraction and Appraisal

All abstracts were screened for eligibility by two independent observers (LJE and MRMJ). In case of disagreement, differences between the observers’ judgements were discussed to seek consensus. In each of the included long-term follow-up papers, data were collected on the number of included adults, the number of coronary complications, and the follow-up time, using data derived from full-text, tables, and overall Kaplan–Meier curves. One observer (LJE) performed a quality assessment of the included papers using the NOS. In patients with coronary complications, data were collected on NYHA class and presence of chest pain when available. Data on the number of adults and duration of follow-up collected from Kaplan–Meier curves were categorized into the following age groups: 18–20 years, 20–25 years, 25–30 years, older than 30 years. It was not possible to identify the censored age for each patient from the Kaplan–Meier curves. Therefore, we were not able to include patients who were censored at the age of 18 and 19 years. Mean follow-up was calculated with the follow-up time derived from Kaplan–Meier curves. 

In cross-sectional anatomical and physiological imaging studies, data on the imaging technique, the number of patients with anatomical high-risk features, perfusion defects, ischemia, and coronary intervention were retrieved. In addition, data of patients with suspicion of myocardial ischemia were collected. Anatomical high-risk features of the proximal coronary arteries following ASO were defined as: acute angle take-off (≤30 degree), interarterial course, ostial stenosis, and/or kinking. These coronary features have been associated with myocardial ischemia in CHD cohorts [14,15].

Suspicion of myocardial ischemia was based on symptoms or ST-segment deviation during exercise tests. Inclusion of imaging studies was based on the median or mean age (≥18 years) of the ASO cohort; therefore, the potential presence of individual patients in this cohort with an age under 18 years could not be excluded. The following data were extracted from case reports: type of examination, type of intervention, presence of chest pain. From pregnancy studies, data on the number of coronary complications, ventricular rhythm disorders, and left ventricular function were collected. The technique and outcome were retrieved from cardiac innervation studies. 

## 3. Results

### 3.1. Study Selection

**Figure 2:**
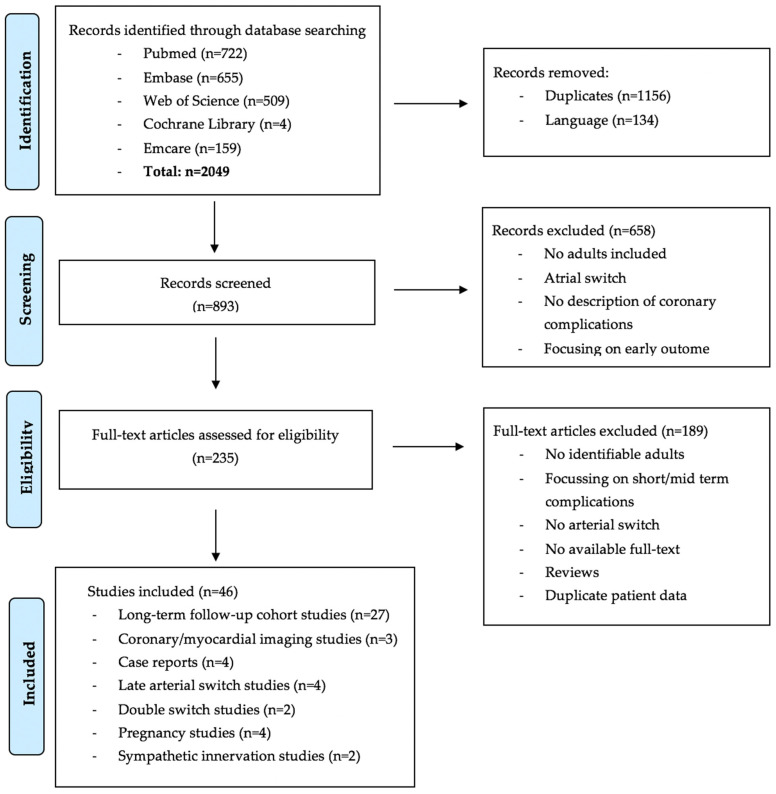
Prisma flow diagram.

A total of 893 studies were identified (Figure 2). The number of excluded studies by abstract screening was 658; most of these reported outcome exclusively in children (<18 years) or focused on atrial switch procedures. The remaining 235 articles were assessed for eligibility by full-text screening. After full-text assessment, 46 studies were included with the following study designs: 27 long-term follow-up cohort studies, 3 cross-sectional imaging studies, and 4 case reports. In addition, studies focusing on the following aspects were included: late arterial switch studies (n = 4), double switch studies (n = 2), pregnancy studies (n = 4), and sympathetic innervation studies (n = 2).

### 3.2. Long-Term Follow-Up Cohort Studies

**Figure 3:**
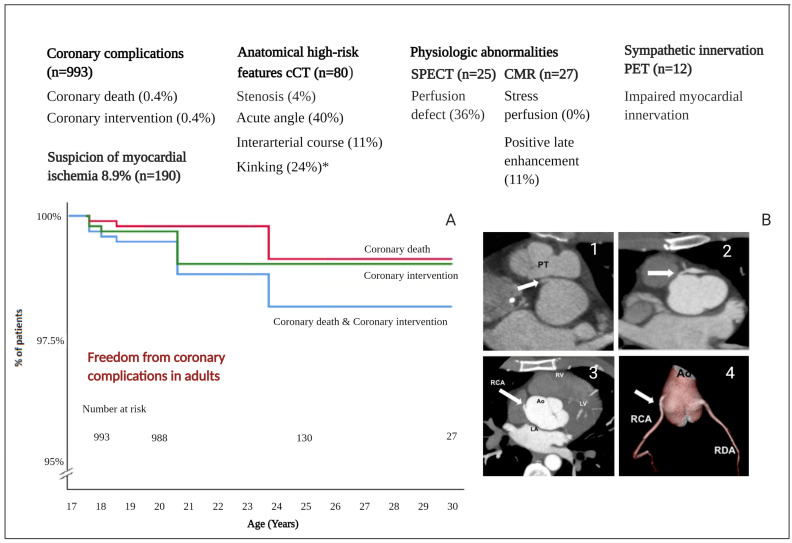
Coronary artery status in adults after ASO. Legend: (**A**). Freedom from coronary complications in adults after ASO. (**B**). Anatomical high-risk features on coronary computed tomography. (B1) Interarterial course of the right coronary artery. (B2) A significant proximal left anterior descending coronary artery stenosis. (B3) Acute angle right coronary artery (axial slice). (B4) Acute angle right coronary artery (three-dimensional reconstruction). Abbreviations: cCT = coronary computer tomography, SPECT = single-photon emission computer tomography, CMR = cardiovascular magnetic resonance, PET = position emission tomography, RDA = Ramus Descendens anterior, RCA = Right Coronary Artery. * only 1 study did describe the presence of kinking (n = 50). Note. CT images B1 and B2 are adapted from ‘Variation in Coronary Anatomy in Adult Patients Late After Arterial Switch Operation: A Computed Tomography Coronary Angiography Study’, by Veltman et al. Ann Thorac Surg, 2013; 96: 1390–1397. Copyright 2021 by Copyright Clearance Center.

A total number of 993 ASO patients were followed for at least 18 years in the long-term follow-up cohort studies [16,17,18,19,20,21,22,23,24,25,26,27,28,29,30,31,32,33,34,35,36,37,38,39,40,41] (Table 1). Figure 3A shows the freedom from coronary complications; the number at risk at 18 years is 993 patients. However, at the age of 25 years the number at risk is only 130 patients, which is explained by the relatively young age of the adult patients described in the included papers. The median follow-up duration into adulthood was 2.0 years (mean 2.8 years). Of the 993 adult ASO patients that were included, a total of 8 (0.8%) coronary-related events were reported. Specifically, four patients (0.4%) underwent a coronary intervention (percutaneous coronary intervention (PCI) or coronary artery bypass grafting (CABG)) for coronary stenosis (n = 2) or occlusion (n = 2). One patient (0.1%) who underwent coronary intervention was asymptomatic. Coronary death was reported in four patients (0.4%): three patients died due to sudden cardiac death, and one patient was found in asystole. All coronary complications are summarized in Table 2. Seventeen out of 192 (8.9%) patients [19,27,42] were suspected to have myocardial ischemia based on symptoms or exercise testing; after additional examinations, 3 out of 17 patients underwent coronary intervention (PCI or CABG).

### 3.3. Coronary Imaging Studies

Two studies [9,42] examined coronary anatomy in adults using routine cCT. The combined results of both studies demonstrated that 4 out of 80 (5%) patients were suspected of coronary stenosis. Three of these patients had no anginal complaints and did not have ischemia at functional testing with single-photon emission computed tomography (SPECT); therefore, no further investigations were performed. In the fourth patient, an angiogram was performed which showed significant proximal luminal narrowing caused by a fibrotic lesion, after which a percutaneous intervention with stent implantation was performed. Other anatomical high-risk features identified by cCT included the following: presence of an acute angle (40%), interarterial course (11%) and proximal kinking (24%). Additional stress SPECT did not show a correlation between perfusion defects and the presence of these high-risk features. Another imaging study in a cohort of 27 ASO adults compared cardiovascular magnetic resonance (CMR) and SPECT under identical physiological circumstances [43]. Nine out of 25 patients (36%) were reported as having perfusion defects on SPECT; however, CMR stress perfusion with dipyridamole was visually normal in all these patients. A summary of coronary abnormalities of all imaging studies (n = 3) is provided in Table 3 and Figure 3.

### 3.4. Case Reports

The literature search identified four case reports [46,47,48,49] (Table 4) in which a successful coronary intervention was reported in adults with a significant coronary lesion. Two patients were diagnosed with a significant stenosis of the left main stem. One patient presented an acute coronary syndrome due to compression of the left main stem. The final patient had subtotal RCA occlusion after pulmonary artery surgery. The presence of chest pain was described in three patients; one patient was asymptomatic.

### 3.5. Pregnancy Studies

Four studies described the outcome in patients during pregnancy [50,51,52,53] (Table 5). 45 patients were followed peri- and post-partum during 73 pregnancies. During these pregnancies, no coronary events were reported. However, two patients (4.4%) had decrease in LV function, and in four patients (9%), heart failure requiring diuretics treatment was reported. Nonsustained ventricular tachycardia was described in four (9%) patients. These outcomes were not associated with coronary events but could partly be attributed to valvular heart disease. Risk factor for adverse outcome included older age at ASO and higher maternal age [50,51,52,53].

### 3.6. Late Arterial Switch and Double Switch

In 12 patients, a late arterial switch operation was performed during adulthood [54,55,56,57] (Table 6). Eleven patients were free from coronary complications during follow-up (range: three months to five years). One patient died five days post-operatively because of low cardiac output syndrome. The outcome of double switch was reported in two adult patients with ccTGA [58,59] (Table 7). Both patients, operated at the age of 14 years and within the first year of life, were free from coronary complications at 5- and 20-year follow-up, respectively. 

### 3.7. Cardiac Sympathetic Innervation

Two studies [60,61] were found on cardiac sympathetic innervation exclusively in adults. Possner and colleagues [60] demonstrated that cardiac sympathetic innervation, measured by [^11^*C*] meta-hydroxyephedrine uptake, was significantly lower in 12 ASO patients compared to 10 healthy individuals, indicating impaired myocardial innervation long-term after ASO. Furthermore, there was no difference in myocardial blood flow (MBF) response after the cold pressor test; however, when corrected for heart rate, the MBF response was lower in ASO patients. This may be explained by an increased release of catecholamines compensating the deficient innervation. The second study [61] investigated myocardial sympathetic innervation in nine ASO patients and nine Rastelli patients by measuring ^11^*C* epinephrine (EPI) retention with positron-emission tomography (PET). In eight ASO patients signs of reinnervation were found, one patient was suspected to have complete denervation (EPI-retention < 7%/min). An association was found between reduced EPI retention and patients undergoing more than one cardiothoracic operation. A summary of findings is provided in Table 8.

## 4. Discussion

This systematic review shows that the number of reported coronary complications in 993 ASO adults is low (0.8%), but the follow-up period after reaching the age of 18 years is limited with a median of only 2.0 years. In our opinion, the limited follow-up duration demonstrates the relevance of this systematic review to provide an overview of current available literature on coronary arterial status of the young adult ASO population because the evidence for current guideline recommendations for coronary follow-up remains limited. Compared to most previous ASO studies in children, the unique aspect of the current systematic review is that it is focused on the coronary status solely in the adult ASO population with a description of late coronary complications, including an overview of both anatomical and physiological coronary imaging studies.

### 4.1. Coronary Imaging Strategy

The review focused on the adult population of each cohort; however, similarly low numbers of coronary interventions in children are reported in two included long-term follow-up cohort studies with a routine coronary imaging strategy and a strategy where coronary imaging was performed on clinical indication. Baruteau and colleagues [22] reported the outcome of a routine coronary imaging strategy with catheterization or cCT in 200 ASO patients; this examination was performed every five years, starting from their fifth year. During a median follow-up of 17 years, coronary stenosis or occlusion was found in 4 patients (2%) at a median age of 9.7 years. Khairy et al. [17] described the coronary outcome in 400 ASO patients during a median follow-up of 18.7 years and reported myocardial infarction in 4 children (1%). In these ASO patients, coronary imaging was performed not on a routine basis but only when clinically indicated.

### 4.2. Imaging Techniques

SPECT can be performed to detect myocardial perfusion defects in patients after ASO. In comparison with the specificity, the sensitivity for detecting coronary stenosis is low [6]. Clinical significance of perfusion abnormalities is unclear and might be related to the operation itself rather than reflecting coronary flow determined by myocardial perfusion [62]. Despite radiation exposure, cCT is a good non-invasive imaging technique alternative for detecting ostial and proximal coronary stenosis compared to gold standard coronary angiography [63]. In the current review, two anatomical imaging studies in adults (n = 80) after ASO demonstrated that anatomical high-risk features, including acute angle, inter-arterial course and kinking were frequently observed at routine cCT. However, additional physiological examination by SPECT at rest and during exercise demonstrated that most of these high-risk features were not associated with significant perfusion defects. A recent systematic review from Morfaw et al. [64] reported a prevalence of any coronary anomaly of 23.0% in ASO patients older than 20 years, although it was not specified how these coronary anomalies were defined. In this review, we focused specifically on the occurrence of coronary complications and coronary high-risk features. The presence of high-risk features may be related to the technical approach of the coronary transfer and not necessarily the original coronary anatomy. This is consistent with Michalak et al. [42] who did not find an association between coronary anomaly and the presence of high-risk features. In their analysis, a proximal coronary stenosis was significantly correlated with a history of complex coronary transfer. Although, the presence of an acute angle was not associated with surgical technique.

Contradictory literature results about the association between coronary features and mortality exists. A meta-analysis by Pasquali and colleagues reported that in almost 2000 ASO patients, all operated before 1999, both an intramural course and single coronary artery anatomy were associated with a higher all-cause mortality rate. However, in a recent study [16], these abnormalities were not found to be a risk factor for death, which may be explained by the improved surgical experience with coronary high-risk features. Data regarding the impact of anatomical high-risk features on myocardial flow is limited, and further investigation is needed [65].

Existing literature on the anomalous aortic origin of a coronary artery (AAOCA) demonstrated that the presence of a high orifice, ostial stenosis, intramural course and length, inter-arterial course, acute angulation and a slit like orifice have been associated with myocardial ischemia [14,15]. The current 2020 ESC guideline for coronary anomalies recommends physical-stress-induced ischemia testing to examine the presence of myocardial ischemia [12]. The clinical significance of the anatomical high-risk features in the ASO population is still undetermined. Progressive neo-aortic root dilatation has been reported in adults after ASO [66]. Due to dilatation of the neo-aortic root in both radial and longitudinal directions, the angle between the coronary artery and aortic wall could decrease progressively during adulthood and may result in coronary stenosis during adult life. Veltman and colleagues [9] reported an association between larger neo-aortic root dimensions and higher coronary take-off in patients with an acute angle, suggesting that individual patient root geometry might play an additional role.

### 4.3. Follow-Up Strategy and Current Findings in Relation to Guidelines Recommendations

To determine the long-term patency of the coronary arteries in this population of young adults, a lifelong follow-up is required. Currently, the 2018 ACC and AHA guidelines for the management of adults with congenital heart disease [11] recommend at least single baseline investigation of the coronary arteries by catheter angiography or cCT. Thereafter, the decision is made on clinical indication. The 2020 ESC guidelines on ACHD [12] recommend cCT when a stenosis is suspected, and they state that a routine non-invasive imaging of coronary arteries with cCT is questionable. Although the current systematic review shows a low number of reported coronary complications, it also exposes that the amount of long-term follow-up data concerning coronary status in adults is still limited. The impact of suture induced fibrosis and atherosclerotic disease on the translocated coronary arties is unclear, and patients with coronary stenosis may be less symptomatic due to decreased innervation post-cardiac surgery. Based on the number of anatomical high-risk features detected by coronary imaging, it seems justified that ASO patients should have at least a baseline assessment of the coronary anatomy with non-invasive CT angiography during young adulthood with additional physiological imaging in patients with high-risk anatomical features or clinical signs of myocardial ischemia.

### 4.4. Limitations

The number of adults extracted from the Kaplan–Meier curves is underestimated because most studies reported the number of patients at risk at 20 years. However, the number of patients censored at the age of 18 and 19 were not reported and therefore could not be included in this systematic review. One explanation for the low number of coronary complications reported during adulthood might be that coronary complications may occur prior to adulthood. In our analysis, these patients were not included because the event occurred during childhood, and these patients were censored before the age of 18 in Kaplan–Meier figures. Secondly, all reported data regarding anatomical high-risk features of the coronary arteries were analysed; however, not all studies described the same coronary features. Thirdly, strategies for coronary follow-up were different between studies. As a consequence of this heterogeneity, coronary interventions may have been performed more frequently in centers with a routine coronary imaging protocol. Finally, all patients after ASO including TGA, DORV and late arterial switch were included in our review because it was not possible to identify individual patients for subgroup analyses.

## 5. Conclusions

The number of reported coronary complications in 993 ASO adults during a median follow-up of 2 years is low (0.8%). Anatomical high-risk features were frequently found by routine coronary imaging with cCT in ASO adults; however, the number of interventions in these patients is low, and the clinical significance of these high-risk features at longer term follow up remains unclear. Based on current findings and in line with the 2018 ACC and AHA guidelines on ACHD, we suggest a baseline assessment of the coronary arteries. Thereafter, an individualized coronary follow-up strategy seems appropriate in young adult patients after the ASO.

## Figures and Tables

**Table 1:** Overview of the extracted studies on coronary complications across different age groups in ASO adults.

			Age Groups (Years)				
			18–20	20–25	25–30	>30	
Author	Patients ≥ 18 Year	Maximum Follow-Up in Adulthood (Yr)	Event/n. at Risk	Event/n. at Risk	Event/n. at Risk	Event/n. at Risk	Newcastle Ottawa Scale
Fricke et al. [16]	183	2	0/183	-	-	-	★★★★★★
Khairy et al. [17]	148	7	0/148	1/28	-	-	★★★★★
Moll et al. [18]	136	7	0/136	0/4	-	-	★★★★★
Kempny et al. [19]	112	20	3/112	1/67	0/20	1/1	★★★★★★
Tobler et al. [20]	60	7	1/60	0/10	-	-	★★★★★★
Santens et al. [21]	52	12	0/52	0/4	0/4	-	★★★★★★
Baruteau et al. [22]	47	7	0/47	0/6	-	-	★★★★★★
Lo Rito et al. [23].	44	2	0/44	-	-	-	★★★★★★
Vida et al. [24]	44	12	0/44	0/1	0/1	-	★★★★★
Oda et al. [25]	40	2	0/40	-	-	-	★★★★
Raissadati et al. [26]	34	12	0/34	0/1	0/1	-	★★★★★★
Ruys et al. [27]	21	2	0/21	-	-	-	★★★★★★
Hörer et al. [19]	17	2	0/17	-	-	-	★★★★★
Shivaram et al. [29]	13	12	0/13	0/3	0/1	-	★★★★★
Lalezari et al. [44]	11	7	0/11	0/2	-	-	★★★★★★
Lim et al. [30]	9	2	0/9	-	-	-	★★★★
Choi et al. [31]	5	7	0/5	0/3	-	-	★★★★★★
Rudra et al. [32]	4	2	0/4	-	-	-	★★★★★★
Gerelli et al. [33]	2	2	0/2	-	-	-	★★★★★★
De Praetere et al. [34]	3	7	0/3	0/1	-	-	★★★★★★
Hayes et al. [45]	2	2	0/2	-	-	-	★★★★★
Hutter et al. [36]	1	2	0/1	-	-	-	★★★★★
Manso et al. [37]	1	2	0/1	-	-	-	★★★★★★
El-Segaier et al. [38]	1	0	1/1	-	-	-	★★★★★★
Gorler et al. [39]	1	2	0/1	-	-	-	★★★★★
Arnaz et al. [40]	1	2	0/1	-	-	-	★★★★
Shim et al. [41]	1	2	0/1	-	-	-	★★★★★
Total	993	median 2.0	5/993	2/130	0/27	1/1	

**Table 2:** Clinical data of ASO patients with coronary complication during adulthood.

Author	Age at ASO	Age at Event (y)	Coronary Anatomy	Chest Pain	NYHA Class	Details
Kempny et al. [19]	-	18	-	-	-	PCI/CABG
Kempny et al. [19]	-	18	-	-	-	PCI/CABG
Tobler et al. [20]	6 days	18	Usual	-	-	Sudden cardiac death
El-Sagaier et al. [38]	4 days	18	Usual	No	-	PCI LCA
Kempny et al. [19]	7 months	19	Cx from RCA	-	III	Sudden cardiac death
Kempny et al. [19]	-	21	-	-	-	PCI/CABG
Khairy et al. [17]	-	24	Single RCA	-	-	Asystole
Kempny et al. [19]	10 y	38	Usual	-	IV	Sudden cardiac death

Abbreviations: NYHA = New York Heart Association, CABG = coronary artery bypass graft, ASO = arterial switch operation, PCI = percutaneous coronary intervention, y = years, LCA = left coronary artery RCA = right coronary. artery, Cx = circumflex.

**Table 3:** Description of findings in cardiac imaging studies in adults after ASO.

						cCT		SPECT		CMR	Angiogram	
Author	N	Age	Common Coronary Anatomy n (%)	Coronary Stenosis n (%)	Inter-Arterialn (%)	Acute Anglen (%)	Kinkingn (%)	Perfusion Defectn (%)	Ischemia n (%)	Ischemia n/Total n	Coronary Stenosis n/Total n	Coronary Intervention(%)
Michalak [40]	50	23	-	3 (6%)	4 (8%)	25 (50%)	12 (24%)	3 (6%)	0 (0%)	-	-	0 (0%)
Veltman [8]	30	22.0	24 (80)	1 (3%)	5 (17%)	7 (23%)	-	3 (14%)	0 (0%)	-	1/1	1 (3.3%)
Tobler [39]	27	21	18 (67)	-	-	-	-	13 (52%)	8 (32%)	0/27	0/1	0 (0%)

Abbreviations: CAD = coronary artery disease, n = number of patients, total n = total number of examined patients, CT = computer tomography, SPECT = single photon emission computed tomography, CMR = cardiovascular magnetic resonance.

**Table 4:** Case reports reporting coronary intervention in ASO adults.

Author	Age	Reason for Intervention	Examination	Chest Pain	Intervention
Quarrie et al. [45]	18 yr	Left main stenosis	SPECT Angio	No	Surgery LIMA-LAD
Stoll et al. [46]	27 yr	ACS due to left main stem compression	CT	Yes	Surgery: defect was reroofed
Ueki et al. [47]	18 yr	subtotal occlusion RCA after patch plasty for PA stenosis	Angiography	Yes	Drug eluting stent
Hamada et al. [48]	24 yr	Ostial stenosis of the left main	CMR & angiography	Yes	Surgery: patch extension

Abbreviations: ACS = acute coronary syndrome, SPECT = single photon emission computed tomography, CMR = cardiovascular magnetic resonance, CT = computer tomography, y = years, LIMA = left internal mammary artery, LAD = left artery descending, PA = pulmonary artery.

**Table 5:** Cardiovascular outcome peri- and post-partum in ASO adults.

				Peripartum			Postpartum		
Author	All pt	Number of Pregnancies	Age	Coronary Event, n	Heart Failure, n	nsVT, n	nsVT, n	Decrease LV Function, n	Heart Failure, n
Stoll, V.M., et al. [49]	15	25	23	0					
Tobler, D., et al. [50]	9	17	22	0			1	2	
Fricke, T.A., et al. [51]	11	17	29	0	1				
Horiuchi, C., et al. [52]	10	14	29	0	0	2	3	0	3

Abbreviations: pt = patient, nsVT = non sustained ventricular tachycardia, n = number of patients, LV = left ventricular.

**Table 6:** Coronary complications during adulthood in patients after late arterial switch.

Author	n. of Adult Patients	Age at ASO	Follow-Up	Coronary Complications
Cetta et al. [53]	1	36 yr	3 m	no
Padalino et al. [55]	1	23 yr	1 yr	no
Watanabe et al. [56]	8	>20 yr	5 yr	no
Maeda et al. [54]	2	18.6 yr & 32.4 yr	-	1 patient died 5 days after ASO due to low cardiac ouput syndrome

Abbreviations: n = number, ASO = arterial switch operation, m = months, yr = year.

**Table 7:** Coronary complications in adults after double switch.

Author	n. of Adult Patients	Age at ASO	Follow-Up	Coronary Complications
Uno et al. [57]	1	14 yr	5 yr	no
Konstantinov et al. [58]	1	within the first year	20 yr	no

Abbreviations: n = number, ASO = arterial switch operation, yr = year.

**Table 8:** Description of findings in cardiac innervation studies in adults after ASO.

				cCT		H2O PET		PET	
				(Ostial)	Myocardial Blood Flow (mL/min/g)			EPI Retention	
Author	All pt	Age	Examination	Stenosis	Rest	Adenosine	Cold Pressor Test	Mean (%/min)	<7%/min
Possner et al. [60]	12	22.5	PET & cTA	0	0.66 ± 0.08	2.23 ± 1.19	0.99 ± 0.20	-	-
Kuehn et al. [61]	9	20.8	PET	-	-	-	-	12.83 ± 1.42	n = 1

Abbreviations: cCT = coronary computed tomography, PET = positron-emission tomography, pt = patient, EPI = epinephrine.

## Appendix A

### Literature Search

Pubmed: (((“Arterial Switch Operation”[Mesh] OR “Arterial Switch Operation”[tw] OR “Arterial Switch Operations”[tw] OR “arterial switch procedure”[tw] OR “arterial switch procedures”[tw] OR “arterial switch repair”[tw] OR “arterial switch surgery”[tw] OR “arterial switch”[tw] OR “Arterial Switch Technique”[tw] OR “Double Switch Operation”[tw] OR “Double Switch Operations”[tw] OR “Double Switch Procedure”[tw] OR “Double Switch Procedures”[tw] OR “Double Switch Technique”[tw] OR “Double Switch”[tw] OR “Jatene Operation”[tw] OR “Jatene Procedure”[tw] OR “Jatene Technique”[tw]) AND (“coronary complications”[tw] OR “coronary complication”[tw] OR “Coronary Disease”[mesh] OR “Coronary Aneurysm”[tw] OR “Coronary Aneurysms”[tw] OR “Coronary Artery Disease”[tw] OR “Coronary Artery Diseases”[tw] OR “Coronary Disease”[tw] OR “Coronary Diseases”[tw] OR “Coronary Occlusion”[tw] OR “Coronary Occlusions”[tw] OR “Coronary Restenosis”[tw] OR “Coronary Restenoses”[tw] OR “Coronary Stenosis”[tw] OR “Coronary Stenoses”[tw] OR “Coronary Thrombosis”[tw] OR “Coronary Thromboses”[tw] OR “Coronary Vasospasm”[tw] OR “Coronary Vasospasms”[tw] OR “Coronary-Subclavian Steal Syndrome”[tw] OR “ischemic event”[tw] OR “ischemic events”[tw] OR “ischaemic event”[tw] OR “ischaemic events”[tw] OR “Myocardial Ischemia”[Mesh] OR “Acute Coronary Syndrome”[tw] OR “Angina Pectoris”[tw] OR “Cardiogenic Shock”[tw] OR “Kounis Syndrome”[tw] OR “Microvascular Angina”[tw] OR “Myocardial Infarct”[tw] OR “Myocardial Infarction”[tw] OR “Myocardial Infarctions”[tw] OR “Myocardial Infarcts”[tw] OR “Myocardial Ischaemia”[tw] OR “Myocardial Ischemia”[tw] OR “Myocardial Reperfusion Injuries”[tw] OR “Myocardial Reperfusion Injury”[tw] OR “Stable Angina”[tw] OR “Unstable Angina”[tw] OR “ischemic disease”[tw] OR “ischemic diseases”[tw] OR “ischaemic disease”[tw] OR “ischaemic diseases”[tw] OR “coronary surgery”[tw] OR “coronary artery surgery”[tw] OR “Coronary Artery Bypass”[Mesh] OR “Coronary Artery Bypass”[tw] OR “Coronary Vessels/surgery”[Mesh] OR “Catheterization”[Mesh] OR “Catheterization”[tw] OR “Catheterisation”[tw] OR Catheter*[tw] OR “Death, Sudden, Cardiac”[Mesh] OR “sudden cardiac death”[tw] OR “Cardiac Sudden Death”[tw] OR “Sudden Cardiac Arrest”[tw] OR “Karoshi Death”[tw] OR “coronary artery anatomy”[tw] OR “coronary artery pattern”[tw] OR “coronary artery patterning”[tw] OR “coronary artery patterns”[tw] OR “coronary outcome”[tw] OR “coronary outcomes”[tw] OR “Chest Pain”[Mesh] OR “thoracic pain*”[tw] OR “thorax pain*”[tw] OR “chest pain*”[tw] OR “Neuralgia”[Mesh] OR “nerve pain*”[tw] OR “neuronal pain*”[tw] OR “neuralg*”[tw] OR “atypical pain*”[tw] OR “Denervation”[Mesh] OR “Denervat*”[tw] OR “reinnervation”[tw] OR “reinnervat*”[tw] OR “re innervat*”[tw] OR “innervation”[Subheading] OR “innervation”[tw] OR “innervat*”[tw] OR “coronary anatom*”[tw] OR “Coronary Vessels/anatomy and histology”[mesh] OR “myocardial perfusion”[tw] OR “Myocardial Perfusion Imaging”[Mesh]) AND (english[la] OR german[la]) AND (“1994/01/01”[PDAT]: “3000/12/31”[PDAT])) OR ((“Arterial Switch Operation”[majr] OR “Arterial Switch Operation”[ti] OR “Arterial Switch Operations”[ti] OR “arterial switch procedure”[ti] OR “arterial switch procedures”[ti] OR “arterial switch repair”[ti] OR “arterial switch surgery”[ti] OR “arterial switch”[ti] OR “Arterial Switch Technique”[ti] OR “Double Switch Operation”[ti] OR “Double Switch Operations”[ti] OR “Double Switch Procedure”[ti] OR “Double Switch Procedure*”[ti] OR “Double Switch Technique”[ti] OR “Double Switch”[ti] OR “Jatene Operation”[ti] OR “Jatene Procedure”[ti] OR “Jatene Technique”[ti]) AND (“outcomes”[tiab] OR “outcome”[tiab] OR “long term”[tiab] OR “longterm”[tiab] OR “experience”[tiab]) AND (english[la] OR german[la]) AND (“1994/01/01”[PDAT]: “3000/12/31”[PDAT]))) NOT (“ablation”[tw] OR “prenatal”[tw] OR “neonates”[ti] OR “neonate”[ti] OR “newborn”[ti] OR “newborns”[ti])

Embase: (((*”Arterial Switch Operation”/OR “Arterial Switch Operation”.ti,ab OR “Arterial Switch Operations”.ti,ab OR “arterial switch procedure”.ti,ab OR “arterial switch procedures”.ti,ab OR “arterial switch repair”.ti,ab OR “arterial switch surgery”.ti,ab OR “arterial switch”.ti,ab OR “Arterial Switch Technique”.ti,ab OR “Double Switch Operation”.ti,ab OR “Double Switch Operations”.ti,ab OR “Double Switch Procedure”.ti,ab OR “Double Switch Procedures”.ti,ab OR “Double Switch Technique”.ti,ab OR “Double Switch”.ti,ab OR “Jatene Operation”.ti,ab OR “Jatene Procedure”.ti,ab OR “Jatene Technique”.ti,ab) AND (“coronary complications”.ti,ab OR “coronary complication”.ti,ab OR exp *”Coronary Artery Disease”/OR “Coronary Aneurysm”.ti,ab OR “Coronary Aneurysms”.ti,ab OR “Coronary Artery Disease”.ti,ab OR “Coronary Artery Diseases”.ti,ab OR “Coronary Disease”.ti,ab OR “Coronary Diseases”.ti,ab OR “Coronary Occlusion”.ti,ab OR “Coronary Occlusions”.ti,ab OR “Coronary Restenosis”.ti,ab OR “Coronary Restenoses”.ti,ab OR “Coronary Stenosis”.ti,ab OR “Coronary Stenoses”.ti,ab OR “Coronary Thrombosis”.ti,ab OR “Coronary Thromboses”.ti,ab OR “Coronary Vasospasm”.ti,ab OR “Coronary Vasospasms”.ti,ab OR “Coronary-Subclavian Steal Syndrome”.ti,ab OR “ischemic event”.ti,ab OR “ischemic events”.ti,ab OR “ischaemic event”.ti,ab OR “ischaemic events”.ti,ab OR exp *”Ischemic Heart Disease”/OR “Acute Coronary Syndrome”.ti,ab OR “Angina Pectoris”.ti,ab OR “Cardiogenic Shock”.ti,ab OR “Kounis Syndrome”.ti,ab OR “Microvascular Angina”.ti,ab OR “Myocardial Infarct”.ti,ab OR “Myocardial Infarction”.ti,ab OR “Myocardial Infarctions”.ti,ab OR “Myocardial Infarcts”.ti,ab OR “Myocardial Ischaemia”.ti,ab OR “Myocardial Ischemia”.ti,ab OR “Myocardial Reperfusion Injuries”.ti,ab OR “Myocardial Reperfusion Injury”.ti,ab OR “Stable Angina”.ti,ab OR “Unstable Angina”.ti,ab OR “ischemic disease”.ti,ab OR “ischemic diseases”.ti,ab OR “ischaemic disease”.ti,ab OR “ischaemic diseases”.ti,ab OR “coronary surgery”.ti,ab OR “coronary artery surgery”.ti,ab OR *”Coronary Artery Bypass Graft”/OR “Coronary Artery Bypass”.ti,ab OR exp *”coronary artery surgery”/OR exp *”Catheterization”/OR “Catheterization”.ti,ab OR “Catheterisation”.ti,ab OR Catheter*.ti,ab OR *”Sudden Cardiac Death”/OR “sudden cardiac death”.ti,ab OR “Cardiac Sudden Death”.ti,ab OR “Sudden Cardiac Arrest”.ti,ab OR “Karoshi Death”.ti,ab OR “coronary artery anatomy”.ti,ab OR “coronary artery pattern”.ti,ab OR “coronary artery patterning”.ti,ab OR “coronary artery patterns”.ti,ab OR “coronary outcome”.ti,ab OR “coronary outcomes”.ti,ab OR *”Thorax Pain”/OR “thoracic pain*”.ti,ab OR “thorax pain*”.ti,ab OR “chest pain*”.ti,ab OR exp *”Neuralgia”/OR “nerve pain*”.ti,ab OR “neuronal pain*”.ti,ab OR “neuralg*”.ti,ab OR “atypical pain*”.ti,ab OR exp *”Denervation”/OR “Denervat*”.ti,ab OR “reinnervation”.ti,ab OR “reinnervat*”.ti,ab OR “re innervat*”.ti,ab OR exp “innervation”/OR “innervation”.ti,ab OR “innervat*”.ti,ab OR “coronary anatom*”.ti,ab OR “myocardial perfusion”.ti,ab OR *”heart muscle perfusion”/OR *”Myocardial Perfusion Imaging”/) AND (english.la OR german.la)) OR ((*”Arterial Switch Operation”/OR “Arterial Switch Operation”.ti OR “Arterial Switch Operations”.ti OR “arterial switch procedure”.ti OR “arterial switch procedures”.ti OR “arterial switch repair”.ti OR “arterial switch surgery”.ti OR “arterial switch”.ti OR “Arterial Switch Technique”.ti OR “Double Switch Operation”.ti OR “Double Switch Operations”.ti OR “Double Switch Procedure”.ti OR “Double Switch Procedures”.ti OR “Double Switch Technique”.ti OR “Double Switch”.ti OR “Jatene Operation”.ti OR “Jatene Procedure”.ti OR “Jatene Technique”.ti) AND (“outcomes”.ti,ab OR “outcome”.ti,ab OR “long term”.ti,ab OR “longterm”.ti,ab OR “experience”.ti,ab) AND (english.la OR german.la))) NOT (“ablation”.ti,ab OR “prenatal”.ti,ab OR “neonates”.ti OR “neonate”.ti OR “newborn”.ti OR “newborns”.ti)

Web of Science: ((ti=(“Arterial Switch Operation” OR “Arterial Switch Operation” OR “Arterial Switch Operations” OR “arterial switch procedure” OR “arterial switch procedures” OR “arterial switch repair” OR “arterial switch surgery” OR “arterial switch” OR “Arterial Switch Technique” OR “Double Switch Operation” OR “Double Switch Operations” OR “Double Switch Procedure” OR “Double Switch Procedures” OR “Double Switch Technique” OR “Double Switch” OR “Jatene Operation” OR “Jatene Procedure” OR “Jatene Technique”) AND ts=(“coronary complications” OR “coronary complication” OR “Coronary Artery Disease” OR “Coronary Aneurysm” OR “Coronary Aneurysms” OR “Coronary Artery Disease” OR “Coronary Artery Diseases” OR “Coronary Disease” OR “Coronary Diseases” OR “Coronary Occlusion” OR “Coronary Occlusions” OR “Coronary Restenosis” OR “Coronary Restenoses” OR “Coronary Stenosis” OR “Coronary Stenoses” OR “Coronary Thrombosis” OR “Coronary Thromboses” OR “Coronary Vasospasm” OR “Coronary Vasospasms” OR “Coronary-Subclavian Steal Syndrome” OR “ischemic event” OR “ischemic events” OR “ischaemic event” OR “ischaemic events” OR “Ischemic Heart Disease” OR “Acute Coronary Syndrome” OR “Angina Pectoris” OR “Cardiogenic Shock” OR “Kounis Syndrome” OR “Microvascular Angina” OR “Myocardial Infarct” OR “Myocardial Infarction” OR “Myocardial Infarctions” OR “Myocardial Infarcts” OR “Myocardial Ischaemia” OR “Myocardial Ischemia” OR “Myocardial Reperfusion Injuries” OR “Myocardial Reperfusion Injury” OR “Stable Angina” OR “Unstable Angina” OR “ischemic disease” OR “ischemic diseases” OR “ischaemic disease” OR “ischaemic diseases” OR “coronary surgery” OR “coronary artery surgery” OR *”Coronary Artery Bypass Graft” OR “Coronary Artery Bypass” OR “coronary artery surgery” OR “Catheterization” OR “Catheterization” OR “Catheterisation” OR Catheter* OR *”Sudden Cardiac Death” OR “sudden cardiac death” OR “Cardiac Sudden Death” OR “Sudden Cardiac Arrest” OR “Karoshi Death” OR “coronary artery anatomy” OR “coronary artery pattern” OR “coronary artery patterning” OR “coronary artery patterns” OR “coronary outcome” OR “coronary outcomes” OR *”Thorax Pain” OR “thoracic pain*” OR “thorax pain*” OR “chest pain*” OR “Neuralgia” OR “nerve pain*” OR “neuronal pain*” OR “neuralg*” OR “atypical pain*” OR “Denervation” OR “Denervat*” OR “reinnervation” OR “reinnervat*” OR “re innervat*” OR “innervation” OR “innervation” OR “innervat*” OR “coronary anatom*” OR “myocardial perfusion” OR *”heart muscle perfusion” OR “Myocardial Perfusion Imaging”) AND la=(english OR german)) OR (ts=(“Arterial Switch Operation” OR “Arterial Switch Operation” OR “Arterial Switch Operations” OR “arterial switch procedure” OR “arterial switch procedures” OR “arterial switch repair” OR “arterial switch surgery” OR “arterial switch” OR “Arterial Switch Technique” OR “Double Switch Operation” OR “Double Switch Operations” OR “Double Switch Procedure” OR “Double Switch Procedures” OR “Double Switch Technique” OR “Double Switch” OR “Jatene Operation” OR “Jatene Procedure” OR “Jatene Technique”) AND ti=(“coronary complications” OR “coronary complication” OR “Coronary Artery Disease” OR “Coronary Aneurysm” OR “Coronary Aneurysms” OR “Coronary Artery Disease” OR “Coronary Artery Diseases” OR “Coronary Disease” OR “Coronary Diseases” OR “Coronary Occlusion” OR “Coronary Occlusions” OR “Coronary Restenosis” OR “Coronary Restenoses” OR “Coronary Stenosis” OR “Coronary Stenoses” OR “Coronary Thrombosis” OR “Coronary Thromboses” OR “Coronary Vasospasm” OR “Coronary Vasospasms” OR “Coronary-Subclavian Steal Syndrome” OR “ischemic event” OR “ischemic events” OR “ischaemic event” OR “ischaemic events” OR “Ischemic Heart Disease” OR “Acute Coronary Syndrome” OR “Angina Pectoris” OR “Cardiogenic Shock” OR “Kounis Syndrome” OR “Microvascular Angina” OR “Myocardial Infarct” OR “Myocardial Infarction” OR “Myocardial Infarctions” OR “Myocardial Infarcts” OR “Myocardial Ischaemia” OR “Myocardial Ischemia” OR “Myocardial Reperfusion Injuries” OR “Myocardial Reperfusion Injury” OR “Stable Angina” OR “Unstable Angina” OR “ischemic disease” OR “ischemic diseases” OR “ischaemic disease” OR “ischaemic diseases” OR “coronary surgery” OR “coronary artery surgery” OR *”Coronary Artery Bypass Graft” OR “Coronary Artery Bypass” OR “coronary artery surgery” OR “Catheterization” OR “Catheterization” OR “Catheterisation” OR Catheter* OR *”Sudden Cardiac Death” OR “sudden cardiac death” OR “Cardiac Sudden Death” OR “Sudden Cardiac Arrest” OR “Karoshi Death” OR “coronary artery anatomy” OR “coronary artery pattern” OR “coronary artery patterning” OR “coronary artery patterns” OR “coronary outcome” OR “coronary outcomes” OR *”Thorax Pain” OR “thoracic pain*” OR “thorax pain*” OR “chest pain*” OR “Neuralgia” OR “nerve pain*” OR “neuronal pain*” OR “neuralg*” OR “atypical pain*” OR “Denervation” OR “Denervat*” OR “reinnervation” OR “reinnervat*” OR “re innervat*” OR “innervation” OR “innervation” OR “innervat*” OR “coronary anatom*” OR “myocardial perfusion” OR *”heart muscle perfusion” OR “Myocardial Perfusion Imaging”)) OR (ti=(*”Arterial Switch Operation” OR “Arterial Switch Operation” OR “Arterial Switch Operations” OR “arterial switch procedure” OR “arterial switch procedures” OR “arterial switch repair” OR “arterial switch surgery” OR “arterial switch” OR “Arterial Switch Technique” OR “Double Switch Operation” OR “Double Switch Operations” OR “Double Switch Procedure” OR “Double Switch Procedures” OR “Double Switch Technique” OR “Double Switch” OR “Jatene Operation” OR “Jatene Procedure” OR “Jatene Technique”) AND ts=(“outcomes” OR “outcome” OR “long term” OR “longterm” OR “experience”))) NOT (ts=(“ablation” OR “prenatal”) OR ti=(“neonates” OR “neonate” OR “newborn” OR “newborns”)) AND la=(english OR german)

Cochrane Library: ((“Arterial Switch Operation” OR “Arterial Switch Operation” OR “Arterial Switch Operations” OR “arterial switch procedure” OR “arterial switch procedures” OR “arterial switch repair” OR “arterial switch surgery” OR “arterial switch” OR “Arterial Switch Technique” OR “Double Switch Operation” OR “Double Switch Operations” OR “Double Switch Procedure” OR “Double Switch Procedures” OR “Double Switch Technique” OR “Double Switch” OR “Jatene Operation” OR “Jatene Procedure” OR “Jatene Technique”):ti,ab,kw AND (“coronary complications” OR “coronary complication” OR “Coronary Disease” OR “Coronary Aneurysm” OR “Coronary Aneurysms” OR “Coronary Artery Disease” OR “Coronary Artery Diseases” OR “Coronary Disease” OR “Coronary Diseases” OR “Coronary Occlusion” OR “Coronary Occlusions” OR “Coronary Restenosis” OR “Coronary Restenoses” OR “Coronary Stenosis” OR “Coronary Stenoses” OR “Coronary Thrombosis” OR “Coronary Thromboses” OR “Coronary Vasospasm” OR “Coronary Vasospasms” OR “Coronary-Subclavian Steal Syndrome” OR “ischemic event” OR “ischemic events” OR “ischaemic event” OR “ischaemic events” OR “Myocardial Ischemia” OR “Acute Coronary Syndrome” OR “Angina Pectoris” OR “Cardiogenic Shock” OR “Kounis Syndrome” OR “Microvascular Angina” OR “Myocardial Infarct” OR “Myocardial Infarction” OR “Myocardial Infarctions” OR “Myocardial Infarcts” OR “Myocardial Ischaemia” OR “Myocardial Ischemia” OR “Myocardial Reperfusion Injuries” OR “Myocardial Reperfusion Injury” OR “Stable Angina” OR “Unstable Angina” OR “ischemic disease” OR “ischemic diseases” OR “ischaemic disease” OR “ischaemic diseases” OR “coronary surgery” OR “coronary artery surgery” OR “Coronary Artery Bypass” OR “Coronary Artery Bypass” OR “Catheterization” OR “Catheterization” OR “Catheterisation” OR Catheter* OR “Death, Sudden, Cardiac” OR “sudden cardiac death” OR “Cardiac Sudden Death” OR “Sudden Cardiac Arrest” OR “Karoshi Death” OR “coronary artery anatomy” OR “coronary artery pattern” OR “coronary artery patterning” OR “coronary artery patterns” OR “coronary outcome” OR “coronary outcomes” OR “Chest Pain” OR “thoracic pain*” OR “thorax pain*” OR “chest pain*” OR “Neuralgia” OR “nerve pain*” OR “neuronal pain*” OR “neuralg*” OR “atypical pain*” OR “Denervation” OR “Denervat*” OR “reinnervation” OR “reinnervat*” OR “re innervat*” OR “innervation” OR “innervation” OR “innervat*” OR “coronary anatom*” OR “coronary anatom*” OR “myocardial perfusion” OR *”heart muscle perfusion” OR “Myocardial Perfusion Imaging”):ti,ab,kw OR (ti=(*”Arterial Switch Operation” OR “Arterial Switch Operation” OR “Arterial Switch Operations” OR “arterial switch procedure” OR “arterial switch procedures” OR “arterial switch repair” OR “arterial switch surgery” OR “arterial switch” OR “Arterial Switch Technique” OR “Double Switch Operation” OR “Double Switch Operations” OR “Double Switch Procedure” OR “Double Switch Procedures” OR “Double Switch Technique” OR “Double Switch” OR “Jatene Operation” OR “Jatene Procedure” OR “Jatene Technique”):ti AND ts=(“outcomes” OR “outcome” OR “long term” OR “longterm” OR “experience”):ti,ab,kw))

Emcare: see Embase.
